# Prognostic value of cardiopulmonary exercise test in children with congenital heart defects

**DOI:** 10.1136/openhrt-2024-002820

**Published:** 2024-08-19

**Authors:** Covadonga Terol, Juliette Hagen, Lukas Rammeloo, Irene M Kuipers, Nicolaas A Blom, Arend DJ ten Harkel

**Affiliations:** 1 Department of Paediatrics, Division of Paediatric Cardiology, LUMC, Leiden, The Netherlands; 2 Department of Paediatrics, Division of Paediatric Cardiology, Amsterdam UMC Locatie AMC, Amsterdam, The Netherlands

**Keywords:** heart defects, congenital, echocardiography, congenital abnormalities

## Abstract

**Background:**

Cardiopulmonary exercise testing (CPET) has an important prognostic value in adults with different congenital heart defects (CHDs) and is a useful tool for risk stratification and clinical decision-making. In this retrospective study, we studied the prognostic value of CPET in paediatric patients with CHD.

**Methods:**

411 CPET performed by paediatric patients with different CHDs were evaluated in this retrospective study. Medical records were reviewed to determine the presence of cardiac events. Participants were classified using the 2018 AHA/ACC guideline for the management of adults with CHD that combines anatomical complexity and current physiological stage.

**Results:**

411 patients with a median age at test of 12 years, 51 patients with simple CHD, 170 patients with moderate complexity CHD and 190 with high complexity CHD underwent CPET. Overall, CPET parameters were lower than the reference values (%predicted VO_2peak_=75% and %predicted oxygen uptake efficiency slope (OUES)=79%), showing worst exercise capacity in the most complex types of CHD (Group III: %predicted VO_2peak_=72% and %predicted OUES=75%). Seventy-one patients presented with cardiac events at a median time from CPET to first event of 28 months. Patients with cardiac events had lower exercise performance as compared with patients without cardiac events as determined by the submaximal variables (%predicted OUES: HR=2.6 (1.5–4.4), p<0.001 and VE/VCO_2_: HR=2.2 (1.4–3.5), p=0.001).

**Conclusion:**

Reduced exercise capacity at young age is related to a higher probability of future cardiovascular events in paediatric patients with CHD. Submaximal exercise variables can be used instead when maximal exercise cannot be achieved.

**WHAT IS ALREADY KNOWN ON THIS TOPIC**

- The prognostic value of cardiopulmonary exercise tests (CPET) in paediatric patients with different congenital heart defects (CHDs) is not well known, whereas in adults it is a useful tool for risk stratification.

**WHAT THIS STUDY ADDS**

- In this retrospective study, we reviewed 411 CPET performed by children with very diverse types of CHD and we found a reduction in exercise capacity in this group of patients. In addition, a lower %predicted value of oxygen uptake efficiency slope and higher value of minute ventilation (VE)/carbon dioxide production (VCO_2_) were related to higher probability of cardiac events at a median time of follow-up of 28 months; 81% of the events consisted of the need of reoperation or reintervention of residual lesions.

**HOW THIS STUDY MIGHT AFFECT RESEARCH, PRACTICE OR POLICY**

- The results of this study highlight the need to routinely perform CPET in paediatric patients with different types of CHDs and provide new perspectives in the decision-making process for reintervention of residual lesions.

## Introduction

 The prevalence of congenital heart defects (CHDs) in children is 9.1 per 1000 live births, amounting to approximately 1.35 million newborns with CHD annually.^1^ CHD encompasses a spectrum from mild to critical malformations, often requiring early surgical intervention, though advancements in diagnosis and treatment have significantly reduced mortality and morbidity.^2 3^ As more patients with CHD reach adolescence and adulthood, optimising quality of life and decision-making regarding reinterventions, such as through assessing exercise capacity, becomes increasingly important.

Cardiopulmonary exercise testing (CPET) is frequently used in patients with CHD as it can objectively evaluate the functional cardiovascular capacity and haemodynamic status of these patients. Several variables obtained by CPET have demonstrated to have an important prognostic value in adults with different CHDs^4^,^7^ and is emerging as a potential tool for risk stratification and clinical decision-making in assessing prognosis and planning interventions.^8^ However, the prognostic value of exercise variables in the paediatric age group is as yet unclear.

In this retrospective study, we aimed to determine the role of CPET in predicting major cardiovascular events (MACEs) in children with different types and degrees of complexity of CHD. We hypothesised that as in adults CPET results in children may help to predict future outcome.

## Materials and methods

### Study population

All patients with a CHD who performed a CPET at the Leiden University Medical Centre (LUMC) between December 2010 and September 2022 and were aged less than 18 years were included in this retrospective study. If patients performed more than one CPET the first available test was used for analysis. The study was approved by the institutional review board and the need for individual consent for this retrospective study was waived.

Follow-up data were collected until November 2022. The medical records were reviewed to determine cardiac events. Cardiac events were defined as overall mortality, the need for heart transplantation, all cardiac-related hospitalisations (including medical management of heart failure, arrhythmia therapies, protein losing enteropathy or plastic bronchitis) and surgical or catheter-based interventions.

To classify the different CHDs in a systematic way, the classification scheme proposed in the 2018 AHA/ACC guideline for the management of adults with CHD^9^ was used. This classification combines anatomical complexity and current physiological stage of the patient to group them in different risk categories and it has shown to improve prediction for cardiac mortality over 15 years in adults as compared with anatomical classification only.^10^ Patients were first classified according to anatomical complexity into low complexity I (eg, atrial and ventricular septal defects or patent ductus arteriosus), moderate complexity II (eg, congenital valve diseases, repaired tetralogy of Fallot or auriculoventricular septal defect) and high complexity III (eg, transposition of the great arteries, valvular atresia or Fontan procedure); and subsequently they were subclassified into four subcategories (A, B, C, D) depending on the physiological stage at the time the CPET was performed. The physiological stage was evaluated reviewing the clinical records of the patients and only information that was within a reasonable period from the CPET was considered.

### Exercise protocol

All patients performed a progressive CPET on an electronically braked cycle ergometer (GE Healthcare eBike Comfort, Freiburg, Germany) connected to a facemask (Hans Rudolph, Kansas City, Missouri, USA) with a flowmeter (Triple V volume transducer) and a computerised gas analyzer (Jaeger MasterScreen CPX, CareFusion GmbH, Hoechberg, Germany; or Vyntus CPX, Vyaire Medical GmbH, Hoechberg, Germany). A breath-by-breath minute ventilation (VE), oxygen uptake (VO_2_), carbon dioxide production (VCO_2_) and respiratory exchange ratio (RER) in 10-s intervals were measured. A 12-lead ECG continuously monitored the heart rate (HR) and blood pressure was determined every 2 min by a sphygmomanometer. A 3-min warm-up phase (unloaded cycling) followed by a continuous incremental bicycle protocol with a work rate increment of 10, 15 or 20 W/min depending on the height (<125 cm, 125–150 cm or >150 cm) according to the Godfrey protocol^11^ was used. The patients had to maintain a pedalling rate between 60 and 80 revolutions/min and were encouraged to perform to exhaustion unless the patient experienced discomfort, changes in the ECG, excessive breathing pattern or otherwise.

RER was defined as the ratio of VCO_2_/VO_2_ and the RER peak (RER_peak_) was calculated as the average of the two highest consecutively achieved RER values in 10 s during peak work rate. An RER_peak_ >1.00 was considered as maximal exercise. The WR peak (WR_peak_) was defined as the maximum work rate achieved and finished (1 min completed) and the %predicted value was calculated.^12^ HR at rest was measured after at least 3 min in a seated position and HR peak (HR_peak_) was calculated as the highest value achieved during at least 10 s in WR_peak_. Then the %predicted value was calculated and ≤90% was considered abnormal.^13^ HR reserve was defined as HR peak minus HR rest.

With the linear regression of VE and VCO_2_ during the entire period of the test the VE/VCO_2_ slope (ventilatory efficiency) and the %predicted values were calculated based on the published reference values.^12^ An absolute value ≥34 was considered abnormal.^13^,^15^

Oxygen uptake efficiency slope (OUES) was calculated by the linear least squares regression of the VO_2_ on the common logarithm of the VE by the equation VO_2_=alog (VE)+b, where the constant ‘a’ is the regression coefficient OUES.^16^ Absolute values, %predicted values and values per body weight were represented. The %predicted (OUES%) was determined using the previous described formulas based on reference normal values adjusted for age and sex.^17^

The maximal oxygen consumption (VO_2peak_), measured in mL/min, was calculated as the average of two highest consecutive achieved VO_2_ values in 10 s during WR_peak_. Reference values were used for calculating the %predicted value (VO_2peak_%) and ≤84% was considered abnormal.^18^ The O_2_pulse is the VO_2_ divided by HR and the maximal O_2_pulse (O_2_pulse_max_) was calculated as the average of the highest two consecutive O_2_pulse values during WR_peak_. The data of Ten Harkel *et al*^12^ were used to calculate the %predicted values and ≤80% were considered abnormal.^13^ Both VO_2peak_ and O_2_pulse_max_ were measured only in patients who achieved RER >1.

### Data analysis

Data analysis was performed using SPSS Statistics software (V.29.0.0.0 IBM SPSS, Chicago, Illinois, USA). Variables were tested for normal distribution using the Kolmogorov-Smirnov or Shapiro-Wilk test when suitable and data were expressed as mean±SD, if normally distributed or as median and IQR if not normally distributed. The exercise test results were expressed relatively to the reference values as % of predicted value (100% would mean equal to reference value) and represented as mean with 95% CI.

One-way analysis of variance with Bonferroni post hoc correction if normally distributed or Kruskal-Wallis test adjusted by the Bonferroni correction for multiple test if not normally distributed were used to assess the differences in CPET variables between the three anatomical and physiological classification groups. The independent-samples t-test or the Mann-Whitney U test, in case of non-normality, were used to assess differences between patients with or without cardiac events.

Kaplan-Meier survival curves and a cox regression, to calculate the HR with the 95% CI, were performed to determine the predictive value of the different CPET variables for cardiac events in the overall group and per anatomical classification group. P values <0.05 were accepted as statistically significant.

## Results

**Figure 1:**
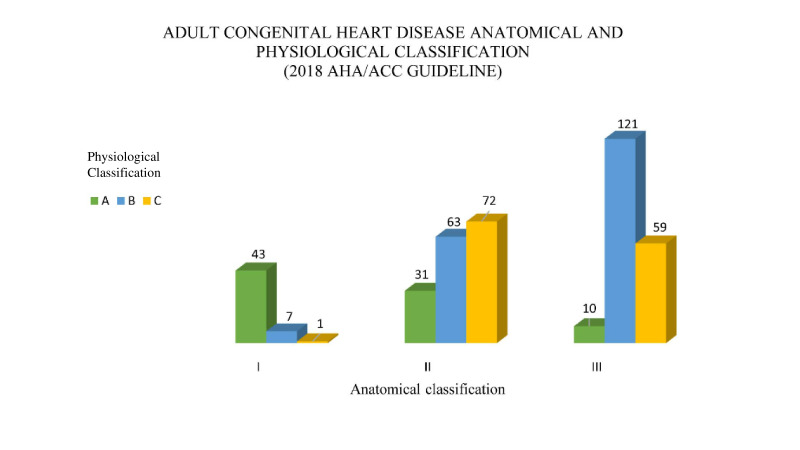
Anatomical and physiological classification according to the 2018 AHA/ACC guideline of the different congenital heart diseases. Classification of patients according to anatomical complexity into three categories: low complexity I, moderate complexity II and high complexity III and subsequently they were subclassified into four subcategories (A in green, B in blue, C in yellow, D not represented as there were no patients in this category) depending on the physiological stage at the time the cardiopulmonary exercise testing was performed.

Four hundred and eleven patients were included in the study with a median (Q1–Q3) age at test of 12 (10–15) years; 43% were female. Figure 1 (and table 1) shows the distribution of the patients with different CHDs according to the 2018 AHA/ACC guideline risk classification. Most of the 51 patients included in the first anatomical group had a good physiological status (A), whereas most of the 170 patients of group II and the 190 patients of group III were in physiological stages B or C (online supplemental table S1). In none of the three anatomical categories were patients in physiological stage D. The clinical assessment to determine the physiological stages of the patients could be assessed at the same time of the CPET in 61%, within 2 months in 29% and within 5 months in 9%. Four patients with an analysis outside this range were excluded. There were no differences in age, sex and size between the three anatomical groups (table 2).

**Table 1:** Types of CHDs according to the 2018 AHA/ACC guideline for the management of adults with CHD

Group I (51 patients)	Group II (170 patients)	Group III (190 patients)
Mild pulmonary stenosis: 3 patients ASD: 10 patients VSD: 38 patients	Tetralogy of Fallot: 40 patients Coarctation of the aorta: 35 patients (3 with VSD) Congenital aortic valve disease: 22 patients AVSD: 16 patients (7 partial and 9 complete) Pulmonary valve stenosis: 13 patients VSD with associated abnormality and/or moderate or greater shunt: 9 patients Anomalous pulmonary venous connection: 7 patients (3 partial and 4 total) Ebstein anomaly: 7 patients Subvalvular aortic stenosis: 5 patients Shone syndrome: 4 patients Anomalous origin of coronary arteries: 4 patients Congenital mitral valve disease: 3 patients Moderate shunt after repaired secundum ASD: 1 patient Absent of pulmonary valve: 1 patient Pulmonary veins stenosis: 1 patient Severe dysplastic tricuspid valve: 1 patient	Fontan procedure (all types): 93 patients TGA (all types): 65 patients Double-outlet ventricle (with biventricular repair): 12 patients Pulmonary atresia (with biventricular repair): 8 patients Truncus arteriosus: 8 patients Cyanotic congenital heart defects: PHT with multiple ASDs: 1 patient Pulmonary stenosis with 1 ½ repair: 3 patients

ASDatrial septal defectAVSDatrioventricular septal defectCHDscongenital heart defectsPHpulmonary hypertensionTGAtransposition of the great arteriesVSDventricular septal defect

**Table 2:** CPET parameters in patients with different congenital heart diseases

	Overall group N=411	In=51 (12.5%)	II n=170 (41.5%)	III n=190 (46%)	P value	P I vs II	P I vs III	P II vs III
Age (years)	12 (10–15)	12 (9–14)	13 (10–15)	12 (10–14)	0.067	0.072	0.479	0.570
Sex (male, %)	234 (57%)	30 (59%)	95 (56%)	109 (57%)	0.921	0.710	0.826	0.776
Medication (yes, %)	113 (28%)	0 (0%)	16 (9%)	97 (51%)	<0.001	0.023	<0.001	<0.001
Weight (kg)	44 (33–57)	43(32–55)	47(35–58)	42(32–55)	0.163	0.592	1	0.233
Height (cm)	156(144–168)	154±15	158(146–169)	154(142–165)	0.167	0.637	1	0.230
BSA (DuBuis)	1.41 (1.17–1.64)	1.38±0.27	1.45 (1.20–1.66)	1.37 (1.14–1.62)	0.149	0.578	1	0.209
SBP_basal_ (mm Hg)	119 (110–134)	115 (108–132)	119 (110–133)	119 (108–134)	0.511	0.775	0.895	1
SBP_peak_ (mm Hg)	164 (147–189)	172±26	163 (146–189)	167±30	0.430	0.656	0.667	1
RER_peak_	1.12±0.10	1.15±0.08	1.12±0.1	1.10±0.1	0.005	0.449	0.010	0.076
RER_peak_ >1 n (%)	373 (91%)	51 (100%)	154 (91%)	168 (89%)	0.046	0.028	0.020	0.483
WR_peak_ (W)	110 (80–160)	120(90–175)	120(90–166)	100 (75–135)	<0.001	1	0.015	<0.001
%predicted	89 (81–97)	100 (95–106)	98 (78–117)	78 (74–81)	<0.001	0.006	<0.001	<0.001
HR_rest_ (bpm)	83±16	82±16.7	83.5±13.7	82±17.5	0.663	1	1	1
HR_peak_ (bpm)	180 (165–189)	186 (176–193)	181(169–190)	173 (162–186)	<0.001	0.261	<0.001	<0.001
%predicted	94 (93–95)	97 (95–99)	96 (94–97)	91 (90–93)	<0.001	0.472	<0.001	<0.001
HR_reserve_ (bpm)	95 (80–110)	108 (93–115)	95.6±17.7	88 (74.5–110)	<0.001	0.039	<0.001	0.057
Submaximal effort parameters								
VE/VCO_2_	30.6 (27.3–34.6)	29.3±4.3	29.1 (26.5–32.1)	32.8 (28.3–37.4)	<0.001	1	<0.001	<0.001
%predicted	105 (103–107)	96 (92–100)	100 (97–103)	112 (108–116)	<0.001	0.600	<0.001	<0.001
OUES (mL/min/log(L/min))	1503 [1213–1876]	1502 [1273–1957]	1576 [1318–1998]	1450 [1156–1799]	0.012	1	0.342	0.004
%predicted	79 (77–81)	86 (80–92)	80 (77–83)	75 (72–79)	0.004	0.794	0.013	0.031
Maximal effort parameters								
VO_2peak_ (mL/min)	1474 [1175–1936]	1639 [1194–2061]	1565 [1301–2034]	1323 [1102–1696]	0.118	0.779	1	1
%predicted	75 (73–78)	83 (76–90)	76 (73–79)	72 (67–77)	<0.001	0.625	0.001	0.004
O_2_pulse_max_ (mL/bpm)	8.7 [7.0–10.8]	8.9 [7.3–11.2]	9.1 [7.3–11.2]	8.2 [6.9–10.2]	0.049	1	0.321	0.066
%predicted	70 (68–72)	78 (68–87)	70 (67–73)	67 (64–71)	0.004	0.476	0.008	0.068

Data shown as mean±SD, median [IQR] or number (%).

The %predicted values are shown as mean (95% CI).

BSAbody surface areaCPETcardiopulmonary exercise testingHR_peak_maximal heart rate at peak exerciseHR_reserve_maximal heart rate-resting heart rateHR_rest_resting heart rateO2pulsemaxmaximal O_2_pulseOUESoxygen uptake efficiency slopeRER_peak_respiratory exchange ratio at peak exerciseSBP_basal_systolic blood pressure at restSBP_peak_systolic blood pressure at peak exerciseVE/VCO_2_ventilatory efficiencyVO_2peak_oxygen uptake at peak exerciseWR_peak_peak work rate

Of the 411 patients, 28% were receiving various medications, with Group III having the highest percentage (table 2). Specifically, 105 patients were on antithrombotic and/or anticoagulant medication. Additionally, nine patients were treated with ACE inhibitors (eight with enalapril and one with captopril). Five patients were on β-blockers (sotalol, labetalol, carvedilol and metoprolol). Two patients received medication to reduce pulmonary pressure (sildenafil and bosentan), and four were using diuretics. Lastly, one patient was treated with flecainide.

Overall, CPET variables in patients with different CHDs are lower than the reference values as demonstrated by the %predicted values (table 2: %predicted VO_2_peak=75% (73–78) and %predicted OUES=79% (77–81)). Group III, with the most complex types of CHDs, showed the worst exercise capacity, with all the %predicted variables lower than the other two anatomical groups (table 2). In groups I, II and III the maximum effort (RER_peak_>1) was reached in 100%, 90% and 89% of patients, respectively. Also, the %predicted values of the WR_peak_ were different between groups, with lower values in patients with more complex CHDs (table 2). There were no other differences between groups I and II.

In groups II and III an analysis of the differences in CPET between the different physiological subgroups was performed (online supplemental table S2). The variables were lower in the subgroup with worst physiological classification in group II but those differences were not observed in group III. Group I was not included in the analysis as most of its patients were in physiological group A.

### Cardiac events

In table 3, the differences in CPET variables between patients who had a cardiac event and those who did not are depicted. Only 13 patients were lost to follow-up (seven from group I, three from group II and three from group III) and 72 patients presented cardiac events; 40 patients in group III, 32 patients in group II and none in group I; with a median time to first event of 28 [11–52] months. In table 4, the different types of cardiac events are depicted. Overall, 30 (42% of the total number of interventions) patients needed a catheter-based intervention and 28 (39%) patients needed cardiac surgery.

**Table 3:** Differences in CPET parameters between patients with cardiac and no cardiac events

	Overall			Group II			Group III		
Overall	No events n=326	Events n=72	P value	No events n=135	Events n=32	P value	No events n=147	Events n=40	P value
HR_peak_ (bpm)	180 (165–189)	173 (162–187)	0.033	181 (168–190)	178 (167–187)	0.262	175 (162–186)	168±21	0.186
%predicted	94 (93–95)	91 (89–94)	0.027	96 (94–97)	94 (91–97)	0.281	92 (90–93)	90 (85–93)	0.144
HR_reserve_ (bpm)	95 (81–110)	89±23	0.079	96±17	93±20	0.427	90 (76–110)	85±25	0.230
Submaximal effort									
VE/VCO_2_	30.2 [27.0–34.1]	32.0 [28.8–37.5]	0.001	29.4±4.2	30.7 [28.0–36.0]	0.080	32.7±6.5	34.6 [29.6–41.7]	0.028
%predicted	103 (101–105)	114 (106–122)	0.004	99 (96–1001)	105 (95–115)	0.200	109 (106–113)	121 (110–132)	0.061
OUES (mL/min/log(L/min))	1537 [1241–1950]	1416±504	0.001	1615 [1335–2030]	1471 [1149–1779]	0.078	1478 [1198–1876]	1320±429	0.014
%predicted	80 (78–83)	71 (66–76)	0.001	82 (78–85)	74 (67–82)	0.058	77 (74–81)	69 (62–75)	0.051
Maximal effort	n=300	n=60		n=125	n=26		n=131	n=34	
VO_2peak_ (mL/min)	1531 [1215–19865]	1259 [1023–1635]	0.009	1568 [1335–2044]	1594±590	0.239	1427 [1121–1843]	1202 [952–1437]	0.003
%predicted	76 (73–78)	69 (64–74)	0.056	77 (73–80)	73 (67–79)	0.243	71 (68–75)	66 (59–73)	0.258
O_2_pulse_max_ (mL/bpm)	8.7 [7.2–10.9]	8.2±3.0	0.007	9.2 [7.5–11.2]	8.7±3.6	0.177	8.4 [7.0–10.8]	7.5±2.0	0.006
O_2_ %predicted	72 (69–74)	62 (60–66)	0.009	71 (68–75)	66 (56–75)	0.271	69 (65–73)	58 (54–63)	0.011

Data shown as mean ± SD, median [IQR] or number (%).

The %predicted values are shown as mean (95% CI).

CPETcardiopulmonary exercise testingHR_peak_maximal heart rate at peak exerciseHR_reserve_maximal heart rate-resting heart rateO_2_pulse_max_maximal O_2_pulseOUESoxygen uptake efficiency slope;VE/VCO_2_ventilatory efficiencyVO_2peak_oxygen uptake at peak exercise

**Table 4:** Types of first cardiac events after CPET

Catheter-based interventions (30 patients 42%)
Melody implantation (3 cases) Stent placement: pulmonary valve and arteries, descending aorta, Fontan tunnel or homograft (13 cases) Balloon dilation: pulmonary valve and arteries, pulmonary veins or descending aorta (8 cases) Vascular plug: collaterals in univentricular patients (12 cases) Closure of Fontan fenestration (3 cases)
Surgery (28 patients 39%)
Homograft implantation (14 cases) Contegra change (1 case) Ross procedure (1 case) RVOT obstruction repair (1 case) Sutureless repair of pulmonary veins (2 cases) Cone procedure (2 cases) Replacement of Fontan (1 case) Subaortic valve obstruction repair (1 case) Aortic root replacement (1 case) Closure of ASD II (1 case)
Rhythm disorders (10 patients 14%)
Catheter ablation (7 cases) Implantable cardioverter defibrillator (3 cases) Pacemaker (2 cases)
Others
Endocarditis (1 case) Pleural-pericardial effusion that needed drainage (1 case) Death (1 case)

Note: In some procedures more than one intervention on was performed (eg, stent implantation and close of a collateral or catheter ablation and implantable cardioverter defibrillator placement).

CPETcardiopulmonary exercise testing

**Figure 2:**
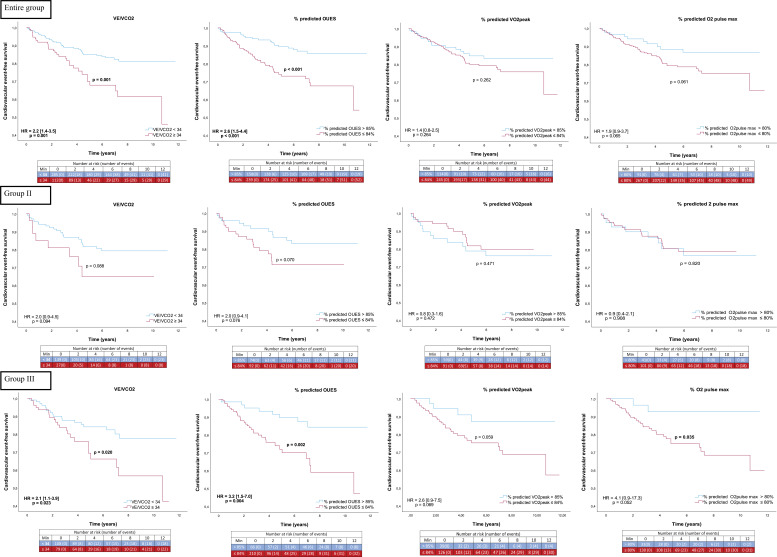
Cardiovascular event-free survival curves. Kaplan-Meier curves for different exercise test parameters in the entire group, group II and group III and their hazard ratios. The analysis was performed using as cut-off points the published reference values (see Material and methods section) and the values considered abnormal were: VE/VCO_2_ >34, %predicted OUES ≤84%, %predicted VO_2peak_ ≤84% and %predicted O_2_pulse ≤80%. O_2_pulse_max_, maximal O_2_pulse; OUES, oxygen uptake efficiency slope; VO_2peak_ oxygen uptake at peak exercise.

Exercise performance was reduced in the group of patients who presented cardiac events in the follow-up period, with lower absolute values and %predicted values of VE/VCO_2_, OUES and O_2_pulse (table 3). There were no differences in group II and in group III only the absolute values were lower in the event group but not the %predicted values (except the O_2_pulse). In the survival analysis (figure 2), %predicted OUES and VO_2peak_ values ≤84% and VE/VCO_2_ ≥34 were related to higher probability of having cardiac events over time (HR=2.6 [1.5–4.4], p<0.001; HR=2.1 [1.2–3.7], p=0.009 and HR=2.2 [1.4–3.5], p=0.001, respectively). In the group analysis, only in group III this association persisted but not in group II.

## Discussion

In the present study, maximal and submaximal exercise capacity were reduced in patients with CHD and this was most prominent in patients with more severe CHD. Lower exercise capacity was associated with a higher likelihood of future cardiovascular events.

CPET tests the integrity of cardiopulmonary, skeletal and autonomic function. Impairment in either of these systems will result in a reduced exercise capacity. In the present study, we found a reduction in the %predicted maximal (VO_2peak_) and submaximal (OUES and VE/VCO_2_) variables. Other studies in patients with CHD with specific cardiac lesions, varying in complexity, also demonstrated reduced exercise capacity in the majority of patients. Even patients after relatively simple CHD surgery, such as coarctation repair, with good long-term outcome show reduced CPET results.^19^ Also patients after surgical VSD closure show reduced exercise capacity as compared with healthy controls, with further deterioration with advancing age.^20 21^ More severely reduced exercise capacity usually is found in the univentricular patients after completion of the Fontan circulation.^6 22^

To compare patient groups with different levels of CHD several classification systems have been developed. The most widely used system is the New York Heart Association (NYHA) classification, that classifies the stages of heart failure in patients with any type of cardiovascular disease ranging from I to IV. A more anatomically based classification is the RACHS-I classification.^23^ This is developed as a perioperative classification especially used for operative mortality. The new classification combining the anatomical and functional characteristics used in adults with CHD^9^ has shown in a group of 629 adult patients that the addition of the physiological stage to the anatomical complexity improved the prediction for cardiac mortality over 15 years.^10^ Although our patients were much younger, to our opinion this classification may be useful in young patients as well as it combines the physiological status of the patients with the anatomical classification. Surprisingly, our study found that CPET variables differed between physiological subgroups only in group II. In contrast, in the group with more severe CHD, CPET variables were similar regardless of physiological status. However, this may have been influenced by the uneven distribution of physiological subtypes within the anatomical groups.

To our knowledge this is the largest group of children with a wide variety of CHD studied with CPET. In such a diverse group we have found that reduced exercise capacity determined by the %predicted values of VE/VCO_2_ and OUES is related to cardiac events (mostly cardiac interventions) irrespectively of the complexity of the CHD. CPET has been extensively used as a predictive tool in adults with different cardiovascular diseases and specifically, in adult patients with CHD.^7 8 24^ In a recent meta-analysis several maximal and submaximal CPET variables have shown to predict MACEs in adults with different CHDs.^4^ Moreover, Wikner *et al* have demonstrated that exercise capacity and diverse group of adults CHD complexity are independently associated with all-cause mortality in patients with CHD.^25^ Data about the prognostic value of CPET in children with CHD are as yet scarce. In Fontan patients’ reduced exercise capacity predicts cardiovascular events after 8 years of follow-up.^6^ Also, Lytrivi *et al* showed that children with CHD and biventricular circulation, exercise testing during heart transplant evaluation identified patients at higher risk of death or earlier clinical deterioration.^26^

In adult’s studies, as mentioned before, CPET can predict MACEs. This term is increasingly been used as outcome in adult studies but its definition is unclear, and includes different endpoints such as cardiovascular death, acute myocardial infarction and stroke and also transplant, initiation of ventricular assist device and cardiac and/or unscheduled hospitalisation.^27^ In the present study, we found that reduced exercise capacity in an unselected population of paediatric patients with CHD also predicts future cardiovascular events, mostly the need of reintervention/reoperation of residual lesion, which opens new perspectives. If exercise capacity is impaired, detailed investigation about underlying mechanisms should be performed, including residual defect, and if possible, they should be treated. This finding also underscores the necessity to evaluate exercise capacity on a regular basis in children with CHD as it can help in the decision-making process for reintervention of residual lesions.

In CPET, the maximal oxygen consumption is usually seen as the gold standard. Most studies that investigate oxygen consumption during exercise use this maximal value. However, in a substantial number of patients a maximal exercise test cannot be performed because of psychological, medical or physical conditions.^28^ In the present study, on average 9% of the patients could not reach an RER >1.0, ranging from 0% in the patients with the simplest lesions up to 11% in the patients with the most severe lesions. Furthermore, in children with a Fontan circulation failure to reach an RER >1.0 can be as high as 20%.^6^ The maximally reached RER in a paediatric population is also age dependent which means a higher failure rate of reaching VO_2peak_ in younger children.^29^ Therefore, submaximal exercise variables have been developed. The VE/VCO_2_ has shown to be a submaximal exercise variable related to mortality in adults with CHD.^8^ More recently the OUES has become more widespread in use,^28^ and has shown to have a good correlation with VO_2peak_ and is a good prognostic marker.^6 30^ In our study, lower %predicted values of both OUES and higher VE/VCO_2_ were related to higher probability of having cardiac events over time. These findings underscore that not only maximal exercise variables have to be assessed but that also submaximal exercise variables have to be taken into account, especially in children and patients with severe underlying disease, in whom maximal effort is not always achieved.

### Limitations

In this retrospective study, all CPET tests that were performed during a certain time period were taken into account. As no strict protocol about when and in which patients a CPET has to be performed was present during that time period, bias may have occurred as in which patients the CPET is performed. However, as the CPET results correlate well with those published in literature we do not think this have influenced our results significantly. While this single-centre design ensures consistency and reliability in the measurements, we recognise the potential limitation in generalisability to broader populations. Future multicentre studies could validate our findings across diverse settings.

The median follow-up period was 7 [3–8] years. If the same results will persist during long-term follow-up is as yet unknown. However, since the number of cardiovascular events increases during adult age it seems unlikely that the effect of previously reduced exercise capacity will disappear.

Most of the patients who underwent CPET were in NYHA classification I or II. If more patients with NYHA III or IV were included, it is likely that more cardiovascular events will be reported, and the prognostic significance of a CPET would probably have been increased.

## Conclusions

Paediatric patients with CHD had overall lower CPET values compared with reference values. Those with the lowest CPET values are more likely to require cardiac interventions over time. When maximal exercise cannot be performed, submaximal exercise variables show similar results and can be used instead. These findings underscore the need to perform CPET on a regular basis in patients with CHD .

## supplementary material

10.1136/openhrt-2024-002820 online supplemental file 1

## Data Availability

Data are available upon reasonable request.
