# Supplementary material for: Prognostic value of cardiopulmonary exercise test in children with congenital heart defects
Source: Open Heart. 2024 Aug 19;11(2):e002820. doi: 10.1136/openhrt-2024-002820 (PMC11337671; doi:10.1136/openhrt-2024-002820)
Supplement: online supplemental file 1 [file openhrt-11-2-s001.pdf]

Table S1. Physiological status. To assess the physiological status, several parameters were considered based on the 2018 AHA/ACC guideline for risk classification. The table highlights the most critical parameter that determined the physiological classification.

PARAMETER	Number of patients (%)
**No altered parameters**	84 (20%)
**Valvular disease**	100 (24%)
**Stenosis of RVOT, PV and/or pulmonary branches**	69 (17%)
**Dilated aortic root according to Detroit Z-scores**	42 (10%)
**Stenosis of LVOT, AoV and/or aortic arch**	24 (6%)
**Ventricular disfunction**	22 (5%)
**Residual shunt**	8 (2%)
**WHO functional class**	7 (2%)
**Hypoxemia/cyanosis**	7 (2%)
**Multifactorial**	43 (11%)

AoV: aortic valve, *LVOT*: left ventricle outflow tract, *PV*: pulmonary valve, *RVOT*: right ventricle outflow tract, *WHO*: world health organization.

Table S2. Differences in CPET parameters between physiological subclassification

Group II		A (N=31)	B (N=63)	C (N=72)	p	p A vs B	p A vs C	p B vs C
**RER_{peak}**		1.15 ± 0.09	1.13± 0.10	1.12 ± 0.09	0.351	1	0.458	1
**RER_{peak} >1**	n (%)	29 (94%)	56 (89%)	66 (92%)	0.633			
**WR_{peak}**	(W)	152 ± 53	120 [90-165]	110 [90-158]	**0.033**	0.344	**0.028**	0.662
	% predicted	103 (96-110)	87 (82-93)	105 (60-151)	**<0.001**	**0.003**	**0.001**	0.831
**HR_{rest}**	(bpm)	89 ± 15	82 ± 15	82 ± 12	0.087	0.134	0.121	1
**HR_{peak}**	(bpm)	182 ± 14	179 ± 14	182 [165-190]	0.568	0.989	0.984	1
	% predicted	97 (94-100)	96 (94-98)	95 (93-97)	0.662	1	1	1
**$HR_{reserve}$**	(bpm)	93 ± 12	97 ± 19	95 ± 19	0.684	1	1	1
**VE/VCO_2**		28.0 ± 3.7	29.6 ± 5.6	30.3 [26.9-33.1]	**0.038**	0.373	**0.033**	0.679
	% predicted	93 (89-97)	99 (95-104)	103 (99-108)	**0.020**	0.214	**0.016**	0.718
**OUES**	(ml/min/log(L/min))	1893 ± 511	1576 [1243-1919]	1503 [1220-1860]	**0.025**	0.121	**0.021**	1
	% predicted	91 (85-96)	80 (74-85)	77 (72-81)	**0.006**	**0.044**	**0.004**	1
**VO_{2peak}**	(ml/min)	1909 ± 505	1575 [1330-2044]	1472 [1227-1971]	**0.043**	0.325	**0.037**	0.884
	% predicted	90 (84-97)	83 (79-88)	80 (75-85)	**0.043**	0.298	**0.037**	0.934
**O_2pulse_{max}**	(ml/bpm)	10.6 ± 3.1	9.1 [7.4-11.0]	9.1 ± 3.2	0.088	0.507	0.084	1
	O2 % predicted	78 (71-85)	71 (65-76)	67 (62-72)	**0.038**	0.113	**0.036**	1
**Group III**		**A (N=10)**	**B (N=121)**	**C (N=59)**	**p**	**p A vs B**	**P A vs C**	**P B vs C**
**RER_{peak}**		1.10 ± 0.08	1.10 ± 0.10	1.10 ± 0.11	0.897	1	1	1
**RER_{peak} >1**	n (%)	10 (100%)	108 (89%)	50 (85%)	0.297			
**WR_{peak}**	(W)	98 [90-205]	100 [75-135]	105 [70-140]	0.478	0.225	0.268	0.899
	% predicted	76 (63-89)	80 (75-84)	74 (68-80)	0.221	1	1	0.253
**HR_{rest}**	(bpm)	81 ± 18	85 [66-96]	81 ± 17	0.704	1	1	1
**HR_{peak}**	(bpm)	171 ± 15	175 [161-187]	170 ± 19	0.770	1	1	1
	% predicted	92 (85-99)	91 (89-93)	91 (88-94)	0.914	1	1	1
**$HR_{reserve}$**	(bpm)	90 ± 28	89 ± 25	92 [79-109]	0.897	1	1	1
**VE/VCO_2**		33.5 ± 7.3	33.7 ± 7.1	31.9 [28.2-36.7]	0.751	1	1	1
	% predicted	115 (98-131)	111 (107-116)	113 (105-120)	0.850	1	1	1
**OUES**	(ml/min/log(L/min))	1883 ± 790	1455 [1186-1741]	1503 ± 568	0.335	0.502	0.424	1
	% predicted	75 (62-89)	77 (73-81)	72 (66-78)	0.336	1	1	0.419
**VO_{2peak}**	(ml/min)	1810 ± 811	1319 [1098-1688]	1323 [1123-1685]	0.917	1	1	1
	% predicted	71 (58-84)	78 (71-84)	71 (66-75)	0.348	1	1	1
**O_2pulse_{max}**	(ml/bpm)	10.1 ± 4.1	8.1 [6.9-10.2]	8.3 [6.8-13.6]	0.847	1	1	1
	O2 % predicted	70 (53-87)	68 (64-73)	64 (59-69)	0.370	1	1	0.485

Data shown as mean ± SD, median [IQR] or number (%). The % predicted values were shown as mean (95% CI). HR_{peak}: maximal heart rate at peak exercise; $HR_{reserve}$: maximal heart rate-resting heart rate; HR_{rest}: resting heart rate; O_2pulse_{max}: maximal O_2 pulse; *OUES*: oxygen uptake efficiency slope; RER_{peak}: respiratory exchange ratio at peak exercise; VO_{2peak}: oxygen uptake at peak exercise; *VE*: ventilatory efficiency; WR_{peak}: peak work rate.
